# Impact of COVID-19 on the work of Spanish dentists: An early response to the pandemic

**DOI:** 10.4317/jced.57941

**Published:** 2021-02-01

**Authors:** Bruno Baracco, Laura Ceballos, Alejandra Llorente, Mª Victoria Fuentes

**Affiliations:** 1 Faculty of Health Sciences, IDIBO Research Group, Rey Juan Carlos University, Madrid, Spain; 2 Private dental practice, Madrid, Spain

## Abstract

**Background:**

This study aimed to assess the quality of the information about COVID-19 that Spanish dentists felt they were provided; their opinion about the actions by Health Institutions; their perception of the risk infection at work; and the security measures implemented to prevent contagion.

**Material and Methods:**

A specific questionnaire was developed and made available online from March 18th to 20th 2020. Dentists working in Spain were invited to answer. Questions were divided in 4 sections: demographic data and professional activity; specific information on COVID-19 and opinion about the decisions taken by Dental Councils and Health Authorities; risk assessment for SARS-CoV-2 in dental practices; and implementation of new ways of working. Chi-square tests were computed (*p*<0.05). 873 dentists answered the survey.

**Results:**

A majority of dentists considered that Health Authorities did not take right decisions during the outbreak (86.37%), and were concerned both about getting infected at work (83.16%) and being a potential carrier to their patients (72.97%). Due to COVID-19, 59.11% of the dentists incorporated new protective measures in the dental practice, 60.17% began dealing with emergencies only and 39.18% stopped working.

**Conclusions:**

Most dentists were worried about infection in their workplaces, particularly women and dentists from the most affected regions by COVID-19. Almost 90% of the participants considered that this pandemic will change the way they provide dental care in the future.

** Key words:**COVID-19, pandemic, disease transmission, dentists´ behavior, dental practice.

## Introduction

A new type of coronavirus named SARS-CoV-2 emerged in Wuhan (China) in December 2019 and it was officially confirmed as the responsible pathogen of COVID-19, a contagious disease, which spread around the world in the following weeks (1-3). In Spain, the first recorded case was identified in the Canary Islands on January 21st 2020, same as in Italy. From February 25th onwards there was a steep increase of registered cases after the first positive tests were recorded in the largest cities, Madrid and Barcelona (4). The fast spreading of the contagion caused authorities to activate the first containment measures at a regional level in the Community of Madrid by March 11th: closure of schools and universities, suspension of leisure centers for the elderly, cessation of visits to nursing homes, and cancellation of mass events. This exact same day, WHO declared COVID-19 to be a pandemic (5).

SARS-CoV-2 easily transmits via respiratory droplets and interpersonal contact as saliva appears to be an important reservoir in both symptomatic and asymptomatic carriers (6-8). Therefore, the generation of numerous droplets and aerosols, which is a characteristic unique to dental practice, presents a high risk of infection, mainly due to the inability of regular protective methods to prevent the virus´s spread (9,10). These findings, along with the fact that COVID-19 was rapidly expanding in the Community of Madrid, to the point of becoming one of the most proportionally affected regions worldwide, forced many dentists into making decisions involving their work. In fact, some dental clinics in Spain, mainly in Madrid, closed their activity by their own volition, prior to the state of alert, which was applied from March 14th.

The state of alert decree was silent about dental clinics and, therefore, there was a general understanding no orders were provided to close them. Catalonia Regional Dental Council issued a recommendation for dental clinics to close, except for urgency treatments, on 13th of March. Three days after, Madrid Regional Dental Council took the same position. On March 20th, the General Dental Council of Spain formally requested to the Government for dental clinics to close, except for emergencies, and determined which cases should be treated. Finally, on April 1st, the Ministry of Health confirmed this approach, which had also been adopted by most of the European Union and countries such as Italy, Germany and the Netherlands.

This work aims to reflect the specific situation suffered in Spain during those days, when no official instructions were issued from the competent Health Authorities but the profession had perceived the risk scenario. As far as we know, no reports focused on the behavior of dentists from any European population in response to COVID-19 pandemic are available in the literature. Therefore, the objectives of this study based on a survey of Spanish dentists were to know: 1) their perception regarding the quality of information about COVID-19 effects in Dentistry and whether they felt that the decisions taken by the competent institutions were appropriate, 2) their awareness of the risk of cross-infection in the dental workplaces, and 3) the security measures implemented by them to prevent contagion on those days and the changes in their working procedures.

## Material and Methods

-Questionnaire survey

A questionnaire conceived for dentists who were working in Spain was specifically designed for this study by the authors and made available online using Google Forms as a system solution. A pilot-testing version was responded to by 12 volunteer dentists who were not involved with the study. After minor adjustments, the final version of the questionnaire was approved to be run online.

Cover information about the objectives of the survey, the University conducting the study and the use of data, was provided to the participants. It was also specified that taking part was voluntary and completing the entire questionnaire would take no longer than 5 min. It was also reassured that all the individual data collected were compliant with the data protection and confidentiality guidelines. The online self-administrated survey was widely accessible and available for 3 days (from the 18th until the 20th of March 2020). Informed consent from all participants was obtained at the beginning of the questionnaire.

The questionnaire was composed by 23 questions which were divided in 4 sections:

1- Demographic data and professional activity.

2- Specific information on COVID-19 and opinion about the decisions taken by Dental Councils and Health Authorities.

3- Risk assessment for SARS-CoV-2 undertaken by dental practices.

4- Potential implementation of new ways of working as a result of COVID-19 epidemic.

-Statistical analysis

A descriptive statistic of the responses obtained was performed, expressing the results as percentages. In addition, chi-square tests were applied to determine the association between demographic data (age, gender, geographical location) and other variables. The level of significance was set at α=0.05. Statistical analysis was performed using Stata 16 (StataCorp, College Station, TX, USA).

## Results

This survey was answered by 873 dentists, three more responded to the questions although did not authorize the use of the data. The respondents did not answer all the questions and these missing values were taken into account for the descriptive exposition of data.

1- Demographic data and professional activity

Descriptive information from the questions of this part of the survey are shown in Table 1. Age of participants ranged from 22-70 years with a mean of 39.2 years. For further comparisons, two groups of regions of Spain were set: most affected regions by COVID-19 (MAR) and less affected ones (LAR).

**Table 1:**
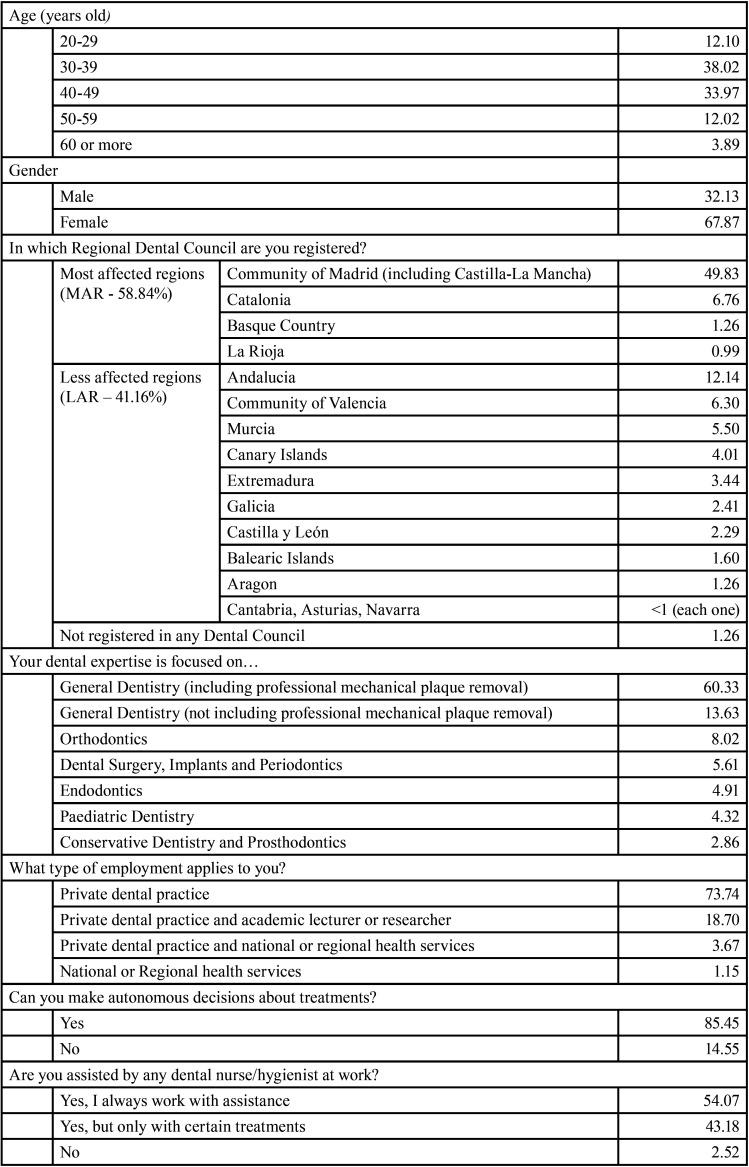
Demographic data and professional activity (%).

2- Specific information on COVID-19 and opinion about the decisions taken by Dental Councils and Health Authorities

From this multiple-selection question it was revealed that the main sources from which dentists obtained information about the effects of COVID-19 in Dentistry were the Regional Dental Council or the General Dental Council of Spain (62.89%). Other channels of information were: “Newspaper, TV and radio” (53.84%), “Websites and social media” (45.70%), “National and local Healthcare Services” (42.04%) and “Workplace” (19.93%).

With respect to the information about the effects of COVID-19 at dental practices provided by the Dental Councils and whether these institutions made the right decision at the right time to prevent the virus’s spread, data are presented in Table 2. For both questions, statistical differences arose for location (*p*<0.001) and gender of participants (*p*=0.011 and *p*=0.006, respectively). A higher percentage of women found that neither the Regional Councils nor the General Dental Council of Spain gave proper information (43.94%) nor took the right decisions (74.01%) than those registered by men (35.88% and 62.29%).

**Table 2:**
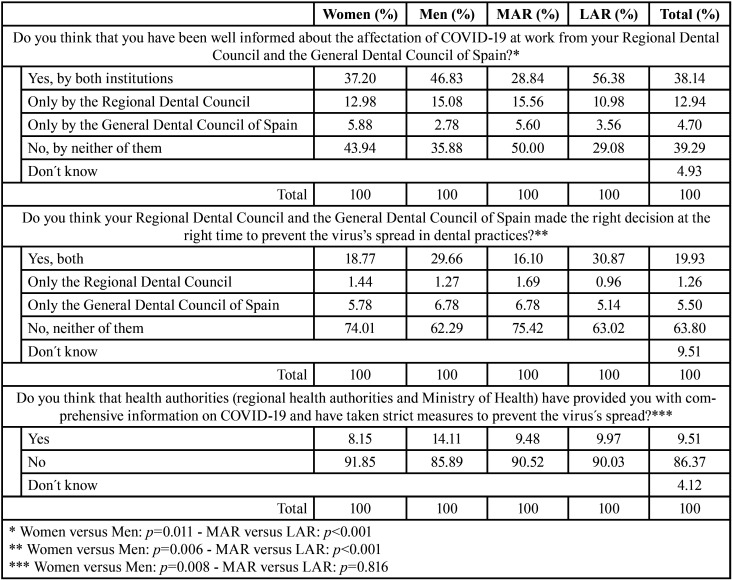
Opinion of dentists regarding the actions taken by Dental Councils and Health Authorities.

When asked how they felt about the specific information on the coronavirus provided in their work, 27.95% stated that they had not received any information, and the same percentage answered “Yes, but it was not comprehensive enough”. Only 16.04% reported that the information received was fully comprehensive. 24.63% of the dentists were the responsible person to provide with the information to their staff and state to have done so, while 1.49% declared they did not provide this information to their staff.

3- Risk assessment for SARS-CoV-2 undertaken by dental practices

According to our results (Table 3), most dentists declared themselves to be concerned about being infected at work by SARS-CoV-2 because they did not have the right protective measures (61.37%) or even though having them (21.81%). Participants who answered not being worried were 16.82%. Statistical differences were detected according to gender (*p*<0.001) and region (*p*=0.03) of the participants: women manifested more worries than men, and dentists from MAR expressed more concern than those from LAR (Table 3). Most participants (87.97%) felt that their chances of being infected by SARS-CoV-2 as a result of their work as a dentist were higher than for other healthcare professions.

**Table 3:**
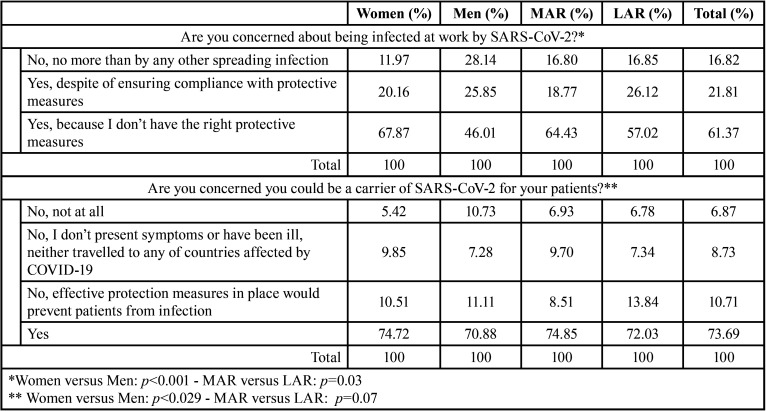
Dentists´ concerns about infection risk in workplaces.

Moreover, majority of participants were concerned about being a carrier of SARS-CoV-2 for their patients (73.69%). Women showed to be significatively more concerned than men (*p*=0.029), while no differences were detected for location (Table 3) and age of the participants (*p*>0.05). Regarding if patients were coming to the dental practice with a strong fear of being infected by coronavirus, most dentists (72.05%) said “Yes” and no differences between regions were recorded (*p*>0.05).

4- Potential implementation of new ways of working as a result of COVID-19 epidemic

Due to COVID-19, 45.59% of dentists incorporated new protective measures in their surgery and for the whole dental practice, 13.52% only in their surgery, 1.72% had not changed their way of working and 39.18% stopped working since becoming aware of the pandemic. In this question, significant differences were found for gender (*p*=0.012) and region (*p*<0.001). Women and dentists from MAR incorporated new protective measures in the surgery in higher proportion than men and dentists from LAR (15.08% versus 9.89%, and 17.19% versus 8.15%, respectively). Instead, no differences regarding the age of the participants arose (*p*>0.05).

Dentists who continued working were also asked if they had halted any type of treatment, and 60.17% declared they were dealing with emergencies only, 19.59% stopped treatments that involved releasing aerosols (including those using high speed), 15.96% were doing the same treatments, and 3.98% stopped doing scale and polish with sprays. When asked why they introduced changes or stopped working, the most replied options for this multiple-selection question were: for social responsibility (75.80%), due to concern about being a carrier of infection that would put the elderly members of my family (49%) or my children (24,97%) at risk, and 22.59% for my own health.

In regard to the introduction of new developments in their way of working, most dentists (76.40%) changed their disinfection protocols since COVID-19 outbreak, while 10.31% had not. Most answered options for this multiple-selection question were: 23.14% increased timing for disinfection, 21.88% increased timing for disinfection and improved surface protection in the surgery, 7.45% improved surface protection in the surgery, 7.22% increased timing for disinfection and upgraded disinfection products (bleach). Moreover, 17.53% of the respondents incorporated all the changes previously described.

Concerning Personal Protective Equipment (PPE), the elements which were most often missing were: FFP2 or FFP3 masks, waterproof gowns and protective glasses (Table 4). In relation to triage for patients, 29.10% of dentists had not incorporated any control system. For those who introduced some sort of triage, most answered options for this multiple-selection question were: 26.80% asking if they presented symptoms (high temperature and respiratory issues), if they had travelled to China, Korea, Italy or other regions affected by COVID-19, and if they had been in contact with positive carriers or people with symptoms; 12.14% asked about symptoms and contact with potential carriers; and 11.23% only about the presence of symptoms. 6.19% began to ask the three questions, and also took patient´s temperature.

**Table 4:**
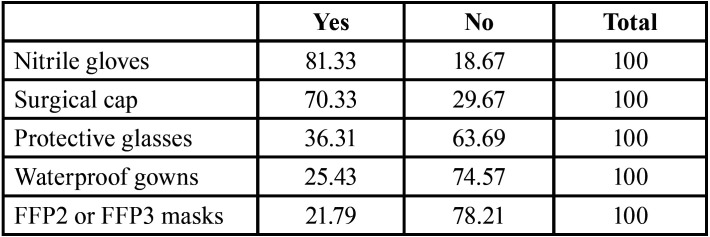
Availability of the different PPE elements by dentists (%).

Most respondents (65.64%) considered that COVID-19 experience will change the way they provide dental care for patients in the future, 23.71% indicated that only for a short time period and 7.33% considered that nothing will change.

## Discussion

The questionnaire was launched through social networks and e-mail. So, the scope of the survey cannot be determined and, by extension, neither can the response rate. The survey was answered by 876 dentists, which is a small sample for the total population of dentists in Spain. However, we would like to remark that the survey was accessible only for 72 hours, inasmuch as the aim of this investigation was to analyze a very short period of time, of just a few days, when COVID-19 expansion was rapidly growing in Spain and no clear recommendations by many Health Authorities had been done. The digital format of the survey and the fact that the authors live and work in Madrid, may have influenced the demographic profile of the participants. In Spain, 60% of all dentists are 40 years old or younger (11) while in this study 53.6% of the participants were 39 years old or younger, so the age distribution seems remarkably similar. With regard to the workplace, 9200 dentists are registered in Madrid Regional Dental Council (including dentists from Castilla-La Mancha. Table 1), which is 25% of the total in Spain. In our study, dentists from this region accounted for 49.83% of the respondents. However, given that Madrid has been one of the most affected areas by COVID-19 in Spain and even in the world (12,13), we understand that the motivation to respond from dentists in Madrid may have been greater than for dentists in other regions.

The distribution of COVID-19 in Spain was not homogeneous throughout the country (13). Before the establishment of the state of alert, cases multiplied rapidly in Madrid, the Basque Country, La Rioja and Catalonia. In fact, Madrid’s decision to close all face-to-face teaching activities was followed by those regions. Therefore, these Autonomous Communities were the only ones to take far-reaching measures to prevent the spread of the coronavirus before any imposition from the national government. We think that these decisions could have had a differentiating effect in terms of the perception of the situation both in the general population and in the dentists working in those territories. This is why the regions were divided into two groups according to their different occurrence of COVID-19.

Regional Dental Councils and the General Dental Council of Spain were the main source of information for dentists regarding the effects of COVID-19 on their profession. However, 39.29% of dentists felt they were not well informed by either of these institutions. Regarding if Dental Councils took timely and effective decisions to stop virus´s propagation, dentists considered that they did not. This opinion was more pronounced in dentists from MAR than those from LAR (75.42% versus 63.02%). However, it could be argued that all dentists made the State Health Authorities more responsible for disinformation and inaction. Dental Councils began to recommend attending only emergencies without waiting for instructions from the Ministry of Health. At the same time, many dentists throughout Spain had already made decisions about their professional activity, when there was very little information about the management of the disease from a dental point of view (9,10). The first official guide from the Ministry of Health for dental clinics was published on 26th May, two and a half months after the first containment measures.

With regard to the perception of risk, a clear majority of participants (83.18%) expressed concern about the possibility of being infected in their work. This is consistent with other studies based on surveys and showing very high fear rates to contagion in dentists from Jordan and Saudi Arabia (14,15). In our study, differences between the answers of dentists from MAR and LAR were significative, as the first ones were more worried about contagion. Also, females showed greater concern than men.

We would like to highlight that very scarce information about specific implications and recommendations for clinical dental care was available when dentists participated in the present study. In this particular regard, investigations from China were already disponible just a few days before (9,10), while other studies from Italy (17-19) and elsewhere (20-22) were published weeks later.

In the present study, most dentists were working in private dental clinics (92.44%) and said that they were autonomous to decide about the treatments performed by them (85.45%). This fact contrasts with other research analyzing the impact of COVID-19 on dentists working in hospitals (15,16). According to our results, 39.18% of dentists stop working since becoming aware of COVID-19 outbreak, a relatively high proportion which matches previous research (15). Only 1.72% of dentists kept working as usual. Between both sides, 59.11% of dentists incorporated new protective measures in their work. Women and dentists from MAR decided to take more protective measures in the surgery compared to men and participants from LAR, respectively. This finding is strongly consistent with the greatest fear of contagion shown by these two groups and seems to be logic since a big number of participants declared themselves capable of making decisions autonomously in their work. In fact, the cabinet is the place where changes ordered by a dentist who does not own the clinic are expected to occur. Additionally, most of dentists stated that they used to work with assistance, something that may allow the introduction or improvement of four-handed Dentistry, a recommended technique to reduce the likelihood of cross-infection (10,17).

In the early stages of the pandemic, 60% of dentists began dealing with emergencies only. Additionally, treatments releasing aerosols were the most avoided procedures by dentists who decided only to halt some of their procedures. Reinforcement of disinfection strategies and setting triage for patients were other initiatives that dentists started without clear guidelines, although, at present, all these strategies are included in the recommended protocols for dental clinics (9,10,17-22). In fact, despite the obvious threat that the situation in Italy must have posed, the impression that the epidemic had not yet reached Spain was reflected in the question to the patients about any recent visit to Italy. This was the general perception in Spain, for example, flights connecting the two countries were suspended on March 11th, just two days before the state of alarm was announced. The lack of proper PPE could also have been a key reason for introducing changes in their work or even reducing/stopping the work in dental clinics (10,17,20). Moreover, one of the most important elements of PPE and most difficult to be purchased (23), FFP2 or FFP3 masks, was the scarcest in the dental clinics in Spain, as only 21.79% of dentists had them (Table 4).

When dentists were asked about which was the main reason that led them to make changes in their work, the most frequently selected reason (75.80%) was “For social responsibility”, which, in our opinion, shows that many Spanish dentists felt the need to reduce or even stop working because of their professional ethics, helping to decrease the number of infections (24). The fact that almost 73% of dentists expressed concerns about being a carrier of SARS-CoV-2 for their patients reinforces this idea.

The impact of COVID-19 in Spain has been severe and is leaving dramatic numbers. To date, October 11th, 33,000 people have deceased and the number of healthcare workers who have been infected is the largest worldwide, according to official Figures from the agency in charge (13). It is unknown whether the various changes that have been introduced in dental clinics since the outbreak of the epidemic will have the capacity to remain in the future. Based on our findings, 65.64% of dentists considered that the way they provide dental care for patients will be altered in a permanent basis. In the past, other spreading infections (mainly hepatitis C and HIV) did promote the incorporation into our routine of new safety measures (25), therefore, the experience of COVID-19 pandemic may represent a new point of no return in terms of protection standards both for dentists and patients.
